# From heart to brain: cognitive potential of propranolol and diltiazem through cholinergic enhancement via butyrylcholinesterase inhibition

**DOI:** 10.3389/fphar.2025.1694610

**Published:** 2025-11-13

**Authors:** Celtia Domínguez-Fernández, Amit Kumar, Rajnish Kumar, Taher Darreh-Shori

**Affiliations:** 1 Division of Clinical Geriatrics, Centre for Alzheimer Research, Department of Neurobiology, Care Sciences and Society, Karolinska Institutet, NEO, Stockholm, Sweden; 2 Department of Pharmaceutical Engineering and Technology, Indian Institute of Technology (B.H.U.), Varanasi, India

**Keywords:** Butyrylcholinesterase (BchE), Acetylcholinesterase (AchE), cholinesterase inhibitors, Alzheimer´s disease (AD), drug repurposing, propranolol, diltiazem

## Abstract

**Background:**

Butyrylcholinesterase (BChE) has emerged as a promising therapeutic target in the treatment of Alzheimer’s disease (AD), particularly in its later stages when acetylcholinesterase (AChE) activity declines. Drug repurposing offers a strategic approach to identify novel BChE inhibitors among existing FDA-approved compounds.

**Objective:**

This study aimed to evaluate the cholinesterase inhibitory potential of propranolol and diltiazem—two widely used cardiovascular drugs—through *in silico* modelling and *in vitro* and *ex vivo* enzyme-inhibition kinetic.

**Methods:**

Molecular docking was performed using AutoDock Vina to assess the binding affinity of propranolol and diltiazem to AChE and BChE. *In vitro* screening and inhibition were measured using a modified Ellman’s assay with human recombinant AChE and plasma-derived BChE. *Ex-vivo* IC_50_ and K_i_ values were determined through kinetic analyses in pooled plasma samples, and inhibition modes were characterized using nonlinear regression models.

**Results:**

Both propranolol and diltiazem selectively inhibited BChE, with minimal activity against AChE. At 100 μM, BChE inhibition exceeded 80% for both compounds, while AChE inhibition was limited to 18% (propranolol) and 2% (diltiazem). Propranolol exhibited a K_i_ of 0.19 µM, comparable to the selective BChE inhibitor ethopropazine (K_i_ = 0.15 µM), and acted as a competitive inhibitor. Diltiazem exhibited a higher K_i_ of 2.3 µM. These effects were observed at concentrations within or near reported brain levels for propranolol, suggesting potential *in vivo* relevance.

**Conclusion:**

Propranolol and diltiazem demonstrate selective BChE inhibition, with propranolol showing potency comparable to established potent BChE inhibitors. Given their established safety profiles and CNS activity, these compounds represent promising candidates for repurposing in the treatment of AD and other cognitive disorders. Further *in vivo* studies are warranted to explore their therapeutic potential.

## 1 Introduction

Acetylcholinesterase (AChE) and butyrylcholinesterase (BChE) are two closely related enzymes that catalyze the hydrolysis of acetylcholine (ACh), a neurotransmitter essential for cholinergic signaling in both the central and peripheral nervous systems. AChE is primarily localized at neuronal synapses, where it rapidly terminates synaptic transmission, whereas BChE is more diffusely distributed, particularly in glial cells and plasma (Darreh-Shori and Soininen, 2010). Cholinergic dysfunction is a hallmark of AD and other dementias, contributing to cognitive decline, behavioural disturbances, and impaired autonomic regulation. Current pharmacological interventions primarily involve cholinesterase inhibitors (ChEIs) such as donepezil, rivastigmine, and galantamine, which aim to enhance cholinergic transmission by inhibiting AChE (Darreh-Shori and Soininen, 2010). While these agents provide symptomatic relief, their clinical efficacy is modest and often limited by gastrointestinal and cardiovascular side effects, largely due to non-selective inhibition of peripheral cholinesterases (Arendt et al., 1984; Giacobini et al., 2022). Moreover, their effectiveness diminishes in the later stages of AD, when AChE levels are significantly reduced and BChE becomes the predominant cholinesterase in the brain (Mesulam M.-M. et al., 2002; Mishra et al., 2019).

Although historically regarded as a secondary enzyme, BChE has gained increasing interest due to several functional distinctions. Notably, while AChE is subject to substrate inhibition at high ACh concentrations, BChE remains highly efficient under these conditions (Giacobini, 2004; Greig et al., 2005). BChE has also been implicated in compensatory mechanisms during neurodegenerative processes, particularly in Alzheimer’s disease (AD). In advanced stages of AD, AChE activity declines to approximately 10%–15% of normal levels, whereas BChE activity remains stable or may even increase up to 120% of baseline (Perry et al., 1978; Giacobini, 2004). This shift suggests a potential compensatory role for BChE in maintaining cholinergic function. Emerging evidence indicates differential interactions between amyloid-β (Aβ) peptides and the two cholinesterases, with distinct consequences for their enzymatic activity (Darreh-Shori et al., 2014; Kumar et al., 2016). For instance, prolonged interaction of AChE with Aβ peptides leads to significant inhibition of AChE activity (Darreh-Shori et al., 2014). In contrast, BChE forms ultrafast catalytic complexes with Aβ peptides, termed BAβACs (BChE–Aβ amyloid-ApoE complexes), which exhibit markedly enhanced hydrolytic activity (Kumar et al., 2016). This hyperactivation of BChE is particularly evident in regions with high Aβ burden, such as near amyloid plaques (Darreh-Shori et al., 2006; Darreh-Shori et al., 2011b; Darreh-Shori et al., 2011a; Darreh-Shori et al., 2014). These BAβACs have been detected in cerebrospinal fluid (CSF) and are hypothesized to gradually accumulate in the AD brain alongside Aβ deposits (Lana et al., 2019). One proposed mechanism suggests that this phenomenon may induce astroglial hyperactivity upon their migration toward Aβ-rich regions. In these areas, astrocytic BChE may acquire ultrafast ACh-hydrolyzing capacity, disrupting the extracellular ACh equilibrium and impairing its regulatory functions in astroglial signaling (Vijayaraghavan et al., 2013; Kumar et al., 2016).

This shift in enzyme dominance highlights the therapeutic potential of targeting BChE, particularly in advanced AD. Selective BChE inhibitors may offer a more tailored therapeutic approach with fewer peripheral side effects. However, few such agents have advanced to clinical application, underscoring the need for novel or repurposed compounds capable of selectively modulating BChE activity while maintaining favourable pharmacokinetic and safety profiles.

Drug repurposing—identifying new therapeutic uses for existing, approved drugs—offers a time- and cost-efficient strategy to accelerate the development of treatments for complex diseases such as AD. In this context, computational screening of FDA-approved compounds has identified several candidates with potential cholinesterase inhibitory activity, including propranolol and diltiazem.

Propranolol, a non-selective beta-adrenergic receptor antagonist, is widely used to treat cardiovascular conditions such as hypertension, angina, and arrhythmia, and is also effective in managing essential tremor and anxiety-related disorders (Srinivasan, 2019; Alharbi et al., 2024). Its ability to cross the blood-brain barrier and modulate central noradrenergic activity has led to its use in neuropsychiatric conditions such as post-traumatic stress disease (PTSD), where it may attenuate the consolidation of traumatic memories and improve cognitive control (Southwick et al., 1999). These effects are mediated through the inhibition of stress-induced noradrenaline release, which otherwise impairs prefrontal cortex function and emotional regulation (Southwick et al., 1999; Arnsten, 2009). Notably, propranolol has demonstrated modest cognitive benefits in clinical studies, suggesting a broader neuroprotective potential (Mahabir et al., 2016).

Other compounds have also been implicated in the treatment of dementia, including calcium channel blockers, which have shown the ability to enhance general cognition. This is attributed to their capacity to reverse amnesia and improve learning and memory in young adult animals (Quartermain et al., 2001). Additionally, they are believed to inhibit Ca^2+^ influx, thereby preventing the formation of amyloid-beta (Aβ) oligomers (Anekonda and Quinn, 2011). Diltiazem, a non-dihydropyridine benzothiazepine calcium channel blocker, is clinically used for angina and other cardiovascular disorders and has demonstrated neurological benefits (Alluri et al., 2023). It has also shown protective effects against traumatic brain injury, cyanide-induced neurotoxicity, and inflammation (Paquet-Durand et al., 2006; Fansa et al., 2009). As previously noted, diltiazem blocks Ca^2+^ entry, thereby disrupting calcium signalling and mitigating amyloid toxicity (Tan et al., 2012). Its favourable safety profile and ability to penetrate the central nervous system (CNS) make it a compelling candidate for repurposing in neurodegenerative diseases (McAuley and Schroeder, 1982).

Despite these promising attributes, the cholinesterase inhibitory properties of propranolol and diltiazem remain underexplored. Given the growing recognition of BChE as a viable therapeutic target in AD and the potential of these drugs to modulate cholinergic function, a systematic evaluation of their inhibitory activity is warranted.

In this study, we investigate the *in vitro* cholinesterase inhibitory effects of propranolol and diltiazem, with a focus on their selectivity for AChE *versus* BChE. By integrating pharmacological profiling with mechanistic insights, we aim to elucidate their potential as repurposed therapeutics for AD and related dementias. This work contributes to the expanding body of research seeking to enhance treatment options for cognitive decline through innovative, mechanism-driven drug repurposing strategies.

## 2 Materials and methods

### 2.1 Study rationale

This study extends previous work on the identification of cholinesterase inhibitors among FDA-approved drugs (Kumar et al., 2017; 2020; Darreh-Shori et al., 2024). A combination of *in silico* molecular docking and *in vitro* enzymatic assays was employed to evaluate the inhibitory potential of propranolol and diltiazem against BChE and AChE. The study also included *ex vivo* enzyme-inhibition kinetic analyses to determine the inhibition constant (K_i_) and mode of enzyme inhibition.

### 2.2 *In silico* molecular docking

To evaluate the interaction phenomenon of the enantiomeric pairs of the drugs propranolol and diltiazem with the BChE enzyme complex, Autodock Vina software was utilized. The crystal structure of receptor enzyme human butyryl cholinesterase (PDB: 1P0I) was downloaded from the RCSB protein data bank in PDB format. The enzyme complex was visualized with the BIOVIA Discovery Studio Client 2024 where all the non-amino acid residues and the water moieties were removed. On the careful visualization, the receptor complex displayed certain missing residues (Glu1, Asp2, Asp3, Asp378, Asp379, Gln455) which was corrected using the model loops options in USCF ChimeraX 1.9 software. The final model which was selected for the docking purpose was the one which displayed the highest negative DOPE score. Then, the best model chosen was subjected to the protein preparation using AutoDock Tools 1.5.7 where the polar hydrogens, Kollmann charges were introduced as well as AD4 type radii was assigned to the receptor. Finally, the prepared protein was saved in PDBQT format. To effectively cover the receptor’s active site the grid box of dimension 26 × 26 × 26 Å was defined. The center coordinates were set around 138.940,500, 116.576,000, 41.126,833 at x, y, and z plane respectively. The enantiomeric structures of propranolol and diltiazem were generated and energy-minimized using the MMFF94 force field in OpenBabel. Docking configuration files were prepared by defining the receptor structure, grid center, and dimensions. Molecular docking was carried out with AutoDock Vina using PERL script on an Ubuntu terminal. The resulting PDBQT output files were visualized in PyMOL to generate receptor-ligand complexes. Binding poses and interaction profiles (2D and 3D) were further analyzed using BIOVIA Discovery Studio Client 2024, which was also employed to prepare docking figures.

### 2.3 Chemical and reagents

Propranolol hydrochloride and diltiazem hydrochloride were obtained from Sigma-Aldrich (St. Louis, MO, United States). The stock solution was prepared at 10 mM in DMSO. Acetylthiocholine iodide (ATC), butyrylthiocholine iodide (BTC), and 5,5′-dithiobis-(2-nitrobenzoic acid) (DTNB) were also purchased from Sigma-Aldrich. Recombinant human AChE (Cat. No. C1682) and pooled human plasma (source of BChE) were used as enzyme sources. The pooled human plasma was prepared as described before (Vijayaraghavan et al., 2013). Briefly, pooled plasma samples were prepared by combining equal volumes from a large cohort of patients clinically diagnosed with mild-to-moderate AD. All samples were collected prior to the initiation of any cholinesterase inhibitor treatment. An equal volume of glycerol was then added to the pooled plasma, thoroughly mixed, and aliquoted into small tubes. These aliquots were stored at -20 °C until used in the assay. Each aliquot was used only once to avoid any interference due to freeze-thaw cycles. All other reagents were of analytical grade.

### 2.4 Cholinesterase inhibition assay

Cholinesterase activity was measured using a modified Ellman’s colorimetric method adapted for high-throughput screening (Kumar et al., 2017; 2020; Darreh-Shori et al., 2024). Assays were conducted in 384-well microplates. Briefly, 20 µL of buffer was initially added to all wells. Subsequently, 20 µL of enzyme solution was added to designated wells, while 20 µL of buffer without enzyme was added to the negative control wells. Each test compound (Hit) was added at 20 µL per well from a 4X working solution (e.g., 4 × 100 µM = 400 µM), using a template transfer plate to ensure efficient and consistent distribution. In the enzyme-control wells, 20 µL of vehicle buffer containing DMSO was added instead of any test compound. This buffer was prepared by diluting pure DMSO in buffer to match the final DMSO concentration in the test wells (i.e., 4% DMSO in the working solution to achieve 1% final concentration in the well). The plate was incubated for approximately 30 min at room temperature with orbital shaking at 100 rpm. The plate reader (Tecan Infinite M1000) was configured for a 384-well transparent plate in absorbance mode at 412 nm, with kinetic reading taken over 10 min at 1-min intervals. Following incubation, 20 µL of Master Mix Buffer (MMB) cocktail was added to each well using a multichannel pipette, and the plate reading was initiated inmediately. The final volume in each well was 80 µL. MMB contained the substrate and DTNB cocktail to reach final concentrations of 0.4 mM DTNB, 5 mM BTC for BChE, and 0.5 mM ATC for AChE. Upon completion of the reading, the data file was saved, enzyme activity rates were extracted, and the data were analyzed. Enzyme activity was calculated from the slope of the linear portion of the absorbance-time curve.

### 2.5 Kinetic analysis and determination of IC_50_ and K_i_

For kinetic studies, a five-point dilution series of propranolol and diltiazem was prepared (ranging from nanomolar to micromolar concentrations). Substrate concentrations were varied in a two-fold serial dilution: BTC (5 mM–156 nM) for BChE and ATC (0.5 mM–31.3 nM) for AChE. Each well received 20 µL of compound or vehicle, 20 µL of substrate, 20 µL of enzyme/DTNB master mix (for all of them 4x working solution in order to reach final concentration), and 20 µL of buffer. Absorbance at 412 nm was measured every 2 min for 15–20 min.

IC_50_ values were calculated by plotting the percentage of residual enzyme activity against the logarithm of compound concentration, followed by fitting the data using a nonlinear regression model with a variable slope in GraphPad Prism 10. This model derives the Hill slope directly from the experimental data rather than assuming a fixed value, thereby minimizing data manipulation and enhancing the accuracy of parameter estimation.

To further elucidate the mechanism of inhibition, inhibition constant (Kᵢ) values and inhibition modes were determined using nonlinear regression models in GraphPad Prism 10. Multiple models were tested, including competitive, noncompetitive, uncompetitive, and mixed inhibition. The optimal model for each compound was selected based on residual analysis and the Akaike Information Criterion (AIC), ensuring robust statistical support. In cases of mixed inhibition, the α parameter was used to characterize the relative affinity of the inhibitor for the enzyme-substrate complex *versus* the free enzyme. The final inhibition mode assigned to each compound was based on the model that provided the best statistical fit and biological plausibility.

All experiments were performed in quadruplicate. Data are presented as mean ± standard deviation (SD).

## 3 Results

### 3.1 *In silico* screening and enantiomer-specific analysis of interactions with cholinesterase enzymes

In our earlier efforts to identify potent inhibitors of ChAT using structure-based drug design approach utilizing SYBYL-X 2.1.1 (Tripos International, St. Louis, MO, USA), we detected several hits that showed a considerable *in silico* affinity (docking score) against BChE and/or AChE (Kumar et al., 2017; 2020; Darreh-Shori et al., 2024). Two of these *in silico* high scoring drugs were diltiazem and propranolol. Sine both of these drugs contain chiral carbons and has multiple enantiomers, a detailed molecular docking analysis of each enantiomer was performed.

**FIGURE 1:**
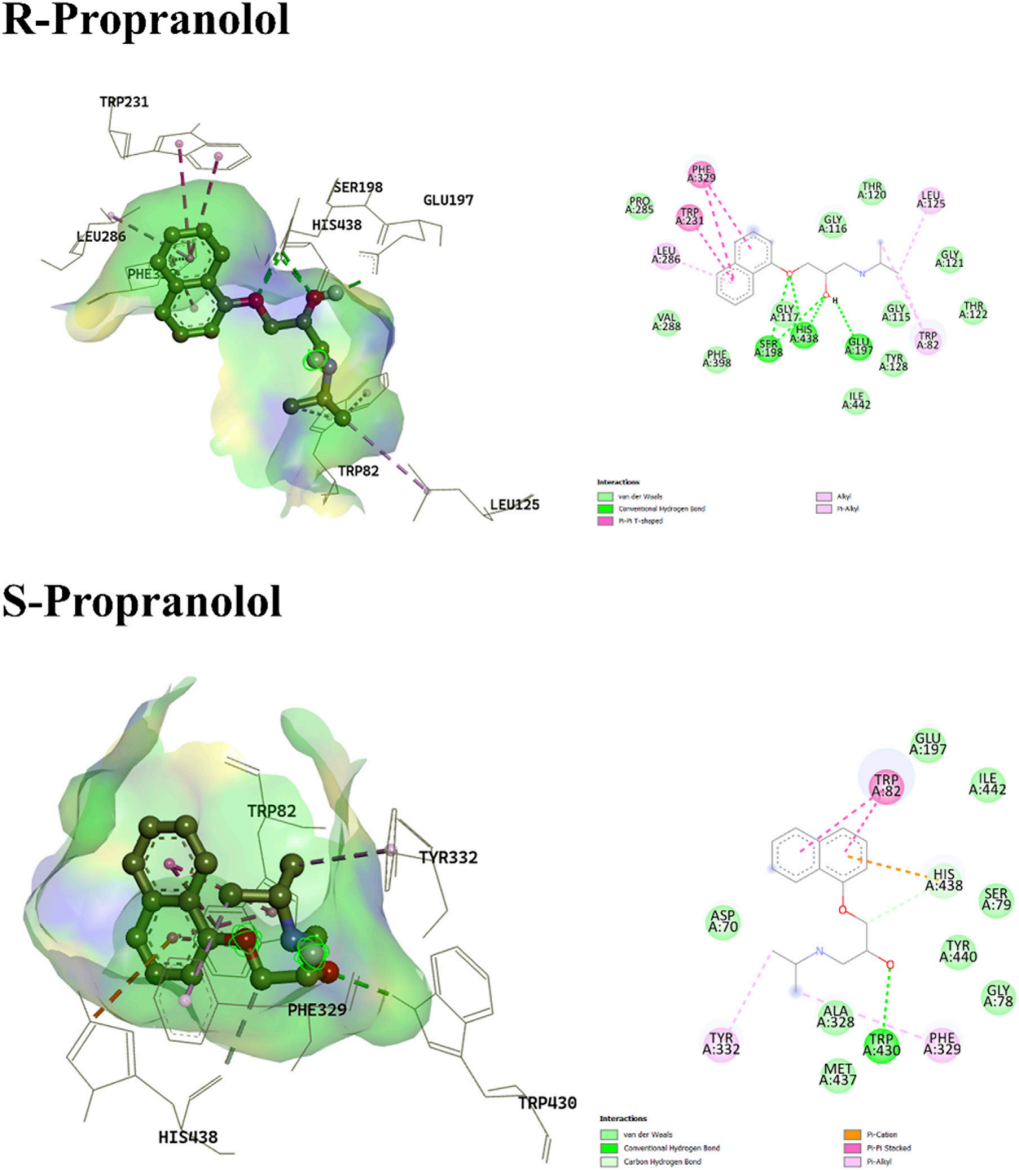
3D and 2D interaction profiles of R-Propranolol and S-Propranolol. Figure displays H-bond interactions with Glu197, Ser198, His438 and Trp430 residues respectively.

The binding affinities and interacting residues are summarized in Table 1 and Figure 1 illustrates the 3D and 2D conformations and binding modes of both S- and R-propranolol enantiomers. The binding energies for propranolol were approximately −7.99 kcal/mol and −7.675 kcal/mol for the R- and S-enantiomers, respectively, indicating a more minimized and stable conformation for the R-enantiomer. Furthermore, the interaction profile of the R-enantiomer appeared more favorable within the active site, exhibiting a higher propensity for hydrogen bond formation with approximately three amino acid residues. The oxygen atoms in the free hydroxyl group and ether linkage interacted with Ser198 and His438, while Glu197 acted as a hydrogen bond acceptor for the hydroxyl group. In contrast, S-propranolol showed weaker hydrogen bonding, interacting only with Trp430. The ether oxygen did not participate in hydrogen bonding, likely due to steric hindrance from surrounding bulky groups. Additionally, His438 displayed distinct interaction patterns with the two enantiomers, forming a π–cation interaction with the S-enantiomer and a hydrogen bond with the R-enantiomer. Alkyl and π–alkyl interactions also differed in terms of the involved residues: Trp82, Leu125, and Leu286 stabilized the R-enantiomer, while Phe329 and Tyr332 contributed to stabilization of the S-enantiomer within the binding pocket.

**TABLE 1:** Binding affinity scores of enantiomers of Propranolol and Diltiazem against BChE.

S.No.	Ligands	Binding affinity (kcal/mol)	Interacting amino acids
1	R-Propranolol*	−7.995	H-bonding: Glu197, Ser198, His438 π – π: Trp231, Phe329 Alkyl or π – Alkyl: Trp82, Leu125, Leu286
2	S-Propranolol*	−7.675	H-bonding: Trp430 π – Cation: His438 π – π Stacked: Trp82 Alkyl or π – Alkyl: Phe329, Tyr332
3	2S,3S-Diltiazem	−8.178	π – π Stacked: Tyr332 Alkyl or π – Alkyl: Trp82, Ala328, Trp430, Met437, Tyr440
4	2R,3R-Diltiazem	−8.486	H-bonding: Ser198, His438 Amide π Stacked: Gly115 Alkyl or π – Alkyl: Leu125
5	2S,3R-Diltiazem	−8.569	H-bonding: Gly116, Gly117, Ser198 π – Cation: His438 π – π Stacked: Tyr332 Alkyl or π – Alkyl: Pro285
6	2R,3S-Diltiazem	−8.824	π – Cation: His438 π – Sulphur: His438 π – π Stacked: Tyr332

* The R-enantiomer of propranolol is dextrorotatory (+) and corresponds to the D-form in older stereochemical nomenclature. Conversely, the S-enantiomer is levorotatory (−) and aligns with the L-form. This distinction is important for readers comparing current data with earlier studies that use the D/L notation, as referenced in (Whittaker et al., 1981; Whittaker et al., 1982).

**FIGURE 2:**
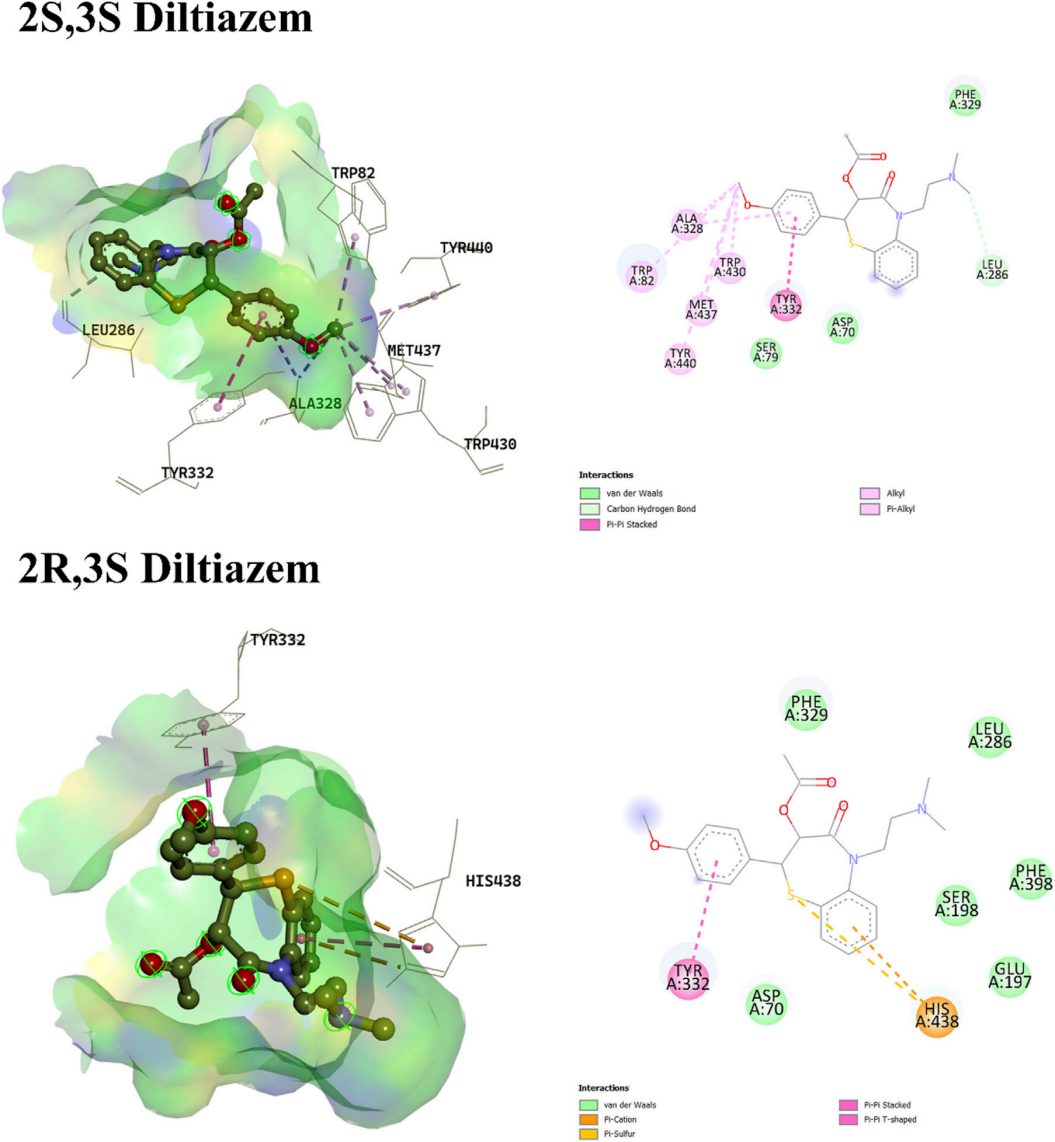
3D and 2D interaction profiles of 2S,3S-Diltiazem and 2R,3S-Diltiazem. Figure displays π–alkyl interactions with Tyr332 and π -sulphur bond with His438.

**FIGURE 3:**
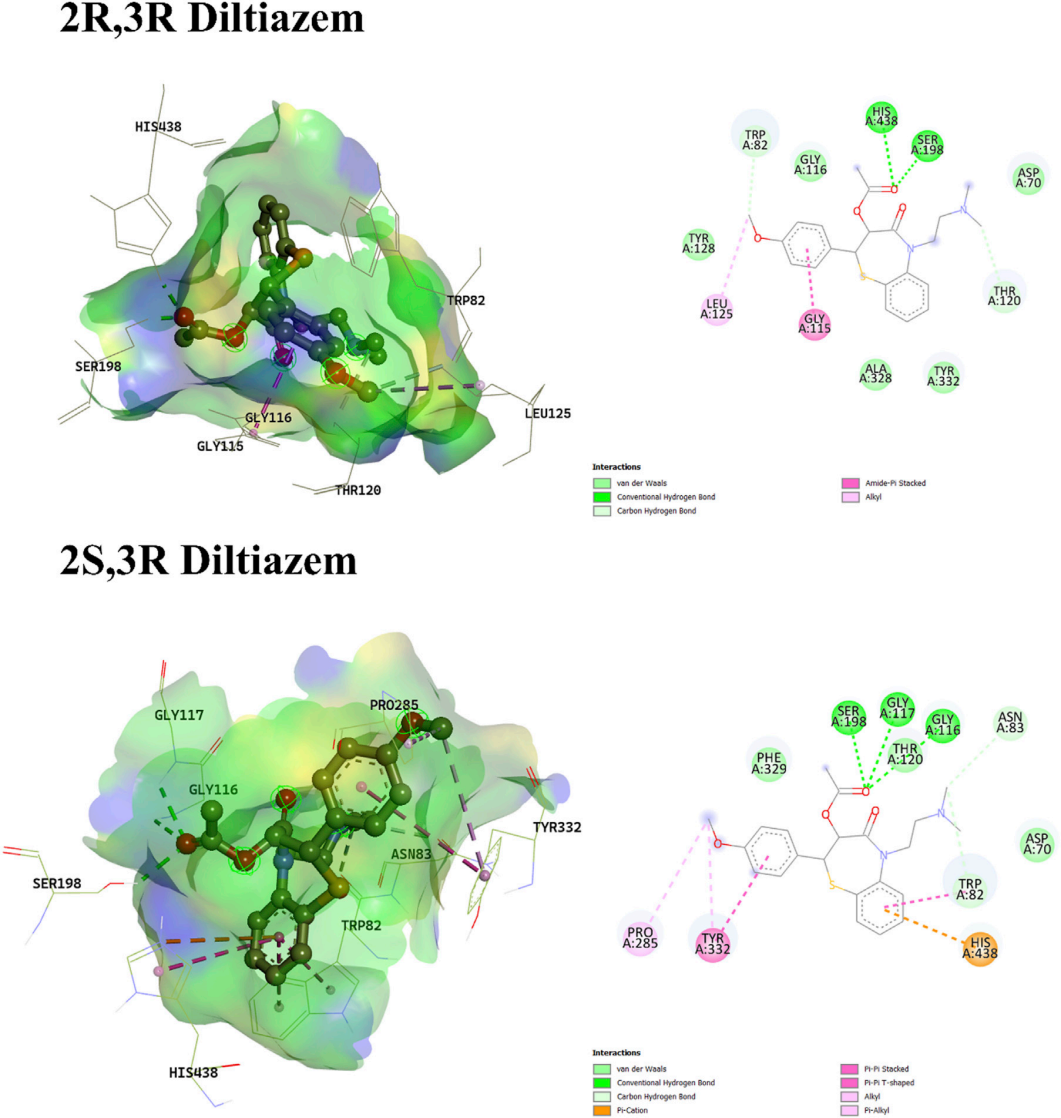
3D and 2D interaction profiles of 2R,3R-Diltiazem and 2S,3R-Diltiazem. Figure displays H-bond interactions with Ser198, His438 and Gly116, Gly117, Ser198 residues respectively.

Regarding diltiazem, which contains two stereocenters, four enantiomers are possible: (2S, 3S), (2R, 3R), (2S, 3R), and (2R, 3S). The binding energies of these enantiomers ranged from −8.178 to −8.824 kcal/mol, with the (2R, 3S) enantiomer exhibiting the most favorable (i.e., most negative) binding score. Enantiomers with identical configurations at both stereocenters showed lower binding affinities compared to those with opposite configurations. The (2S, 3S) and (2R, 3S) enantiomers lacked hydrogen bond interactions, whereas the (2R, 3R) and (2S, 3R) isomers formed hydrogen bonds with Ser198 and His438 (for 2R, 3R), and Gly116, Gly117, and Ser198 (for 2S, 3R). The hydrogen bonding in the latter two enantiomers was attributed to the orientation of the ester group’s carboxylic oxygen toward the binding pocket. In contrast, the carboxylic oxygen in the former enantiomers was oriented away from the binding residues, preventing hydrogen bond formation, as shown in Figures 2, 3, respectively. A unique interaction was observed for the (2R, 3S) enantiomer, where the sulfur atom of the benzothiazepine ring engaged with the π-cloud of His438, contributing to the stabilization of its conformation. Moreover, π–cation interactions with His438 were also noted in enantiomers with differing configurations at both stereocenters.

It is important to note that docking scores do not always correlate with experimental binding affinities, as docking algorithms typically generate multiple binding poses, and accurately identifying the pose that most closely resembles the bioactive conformation remains a significant challenge (Pantsar and Poso, 2018).

### 3.2 *In vitro* screening against cholinesterase enzymes

**FIGURE 4:**
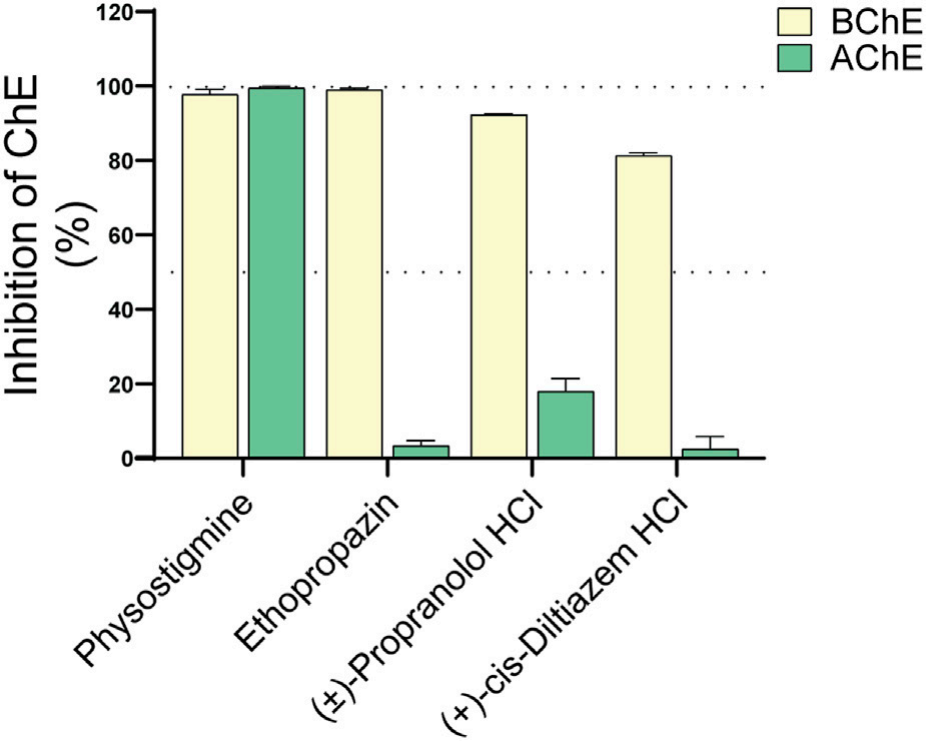
Inhibitory effects of propranolol and diltiazem on cholinesterase activity. Each compound was tested at a single concentration against AChE and BChE. Results are expressed as the percentage of enzyme inhibition relative to the vehicle control. Both propranolol and diltiazem exhibited over 80% inhibitory activity against BChE. In the case of propranolol, this effect was comparable to that of physostigmine and ethopropazine, both well-established BChE inhibitors. In contrast, propranolol showed only modest inhibition of AChE, while diltiazem had no detectable effect on this enzyme. Data are presented as mean ± standard deviation (SD).

Therefore, propranolol and diltiazem were initially screened *in vitro* at a single concentration (100 µM) to assess their inhibitory effects on human AChE and plasma-derived BChE. Both compounds exhibited strong inhibition of BChE, with inhibition rates exceeding 80%. In contrast, their effects on AChE were minimal, with propranolol and diltiazem inhibiting approximately 18% and 2% of AChE activity, respectively. Two reference compounds were included as controls: physostigmine, a non-selective cholinesterase inhibitor that nearly completely inhibited both enzymes, and ethopropazine, a selective BChE inhibitor that showed no inhibition of AChE (Figure 4).

### 3.3 Determination of IC_50_ values

**FIGURE 5:**
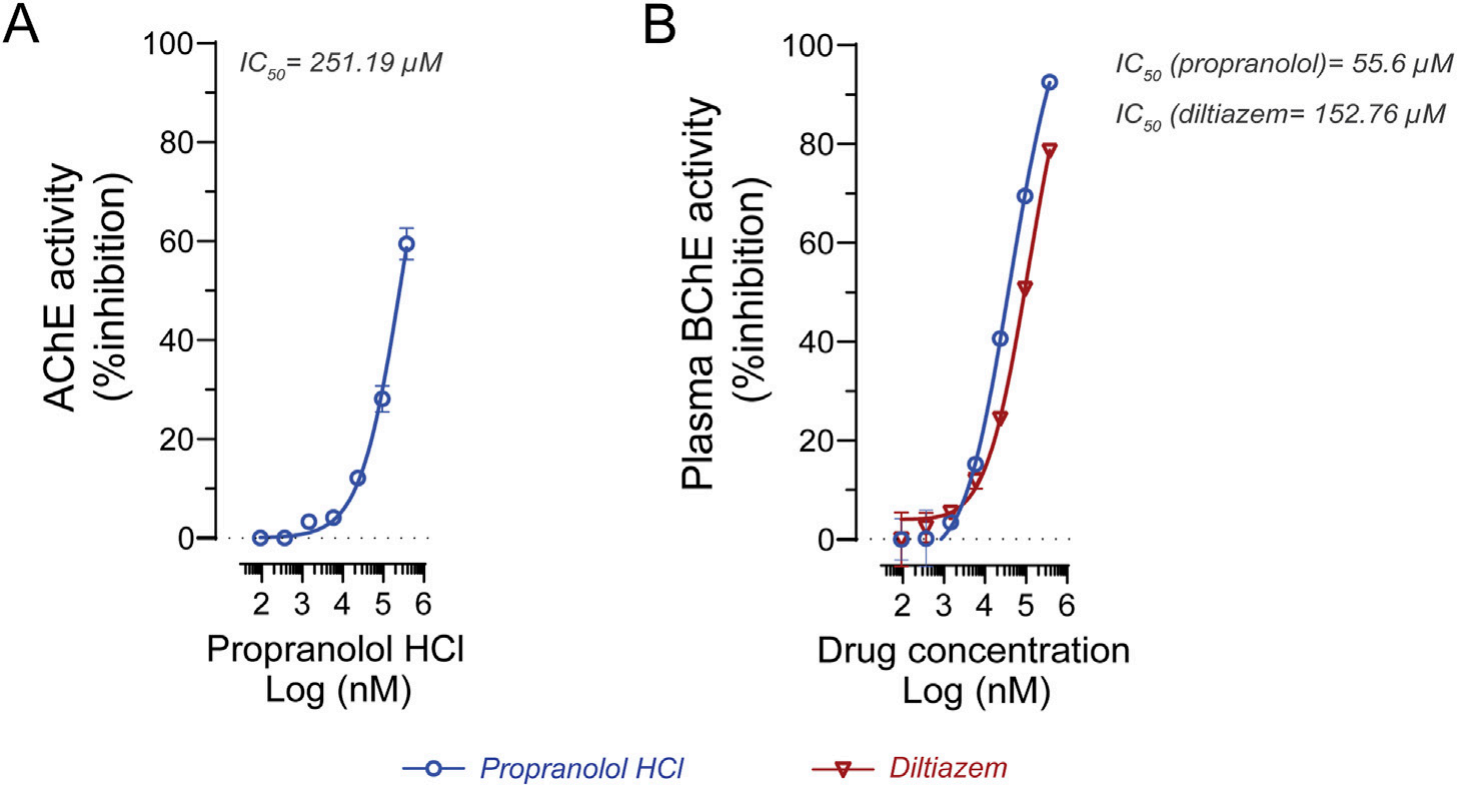
*In vitro* evaluation of propranolol and diltiazem against human cholinesterase. **(A)** IC_50_ determination of propranolol against human AChE yielded a value of 251.19 μM. **(B)** IC_50_ analysis of propranolol and diltiazem against human BChE resulted in values of 55.6 μM and 152.76 μM, respectively. Experiments were conducted using 0.5 mM acetylcholine and 5 mM butyrylcholine as the preferred substrates for AChE and BChE, respectively. Enzyme activity is expressed as a percentage relative to the vehicle control. Data are presented as mean ± standard deviation (SD). IC_50_ values were calculated using GraphPad Prism 9.

To further characterize the inhibitory potency, IC_50_ values were determined under substrate-saturating conditions (Table 2). For AChE, propranolol exhibited an IC_50_ of 251.19 µM using 0.5 mM ATC as substrate (Figure 5A). Due to its negligible inhibition of AChE, diltiazem was not further evaluated for IC_50_ determination against this enzyme. For BChE, IC_50_ values were determined using 5 mM BTC as substrate. Propranolol showed an IC_50_ of 55.6 µM, while diltiazem exhibited an IC_50_ of 152.76 µM (Figure 5B), confirming their preferential inhibition of BChE over AChE since calculated IC_50_ were significantly lower.

**TABLE 2:** IC_50_ and Ki of all tested compounds.

	IC_50_ (AChE)	IC_50_ (BChE)	Ki (BChE)
Propranolol	251.19 µM	55.6 µM	0.19 µM
Diltiazem		152.76 µM	2.3 µM
Physostigmine		21.18 µM	0.51 µM
Ethopropazine		2.81 µM	0.15 µM

### 3.4 Kinetic analysis and determination of K_i_ values

**FIGURE 6:**
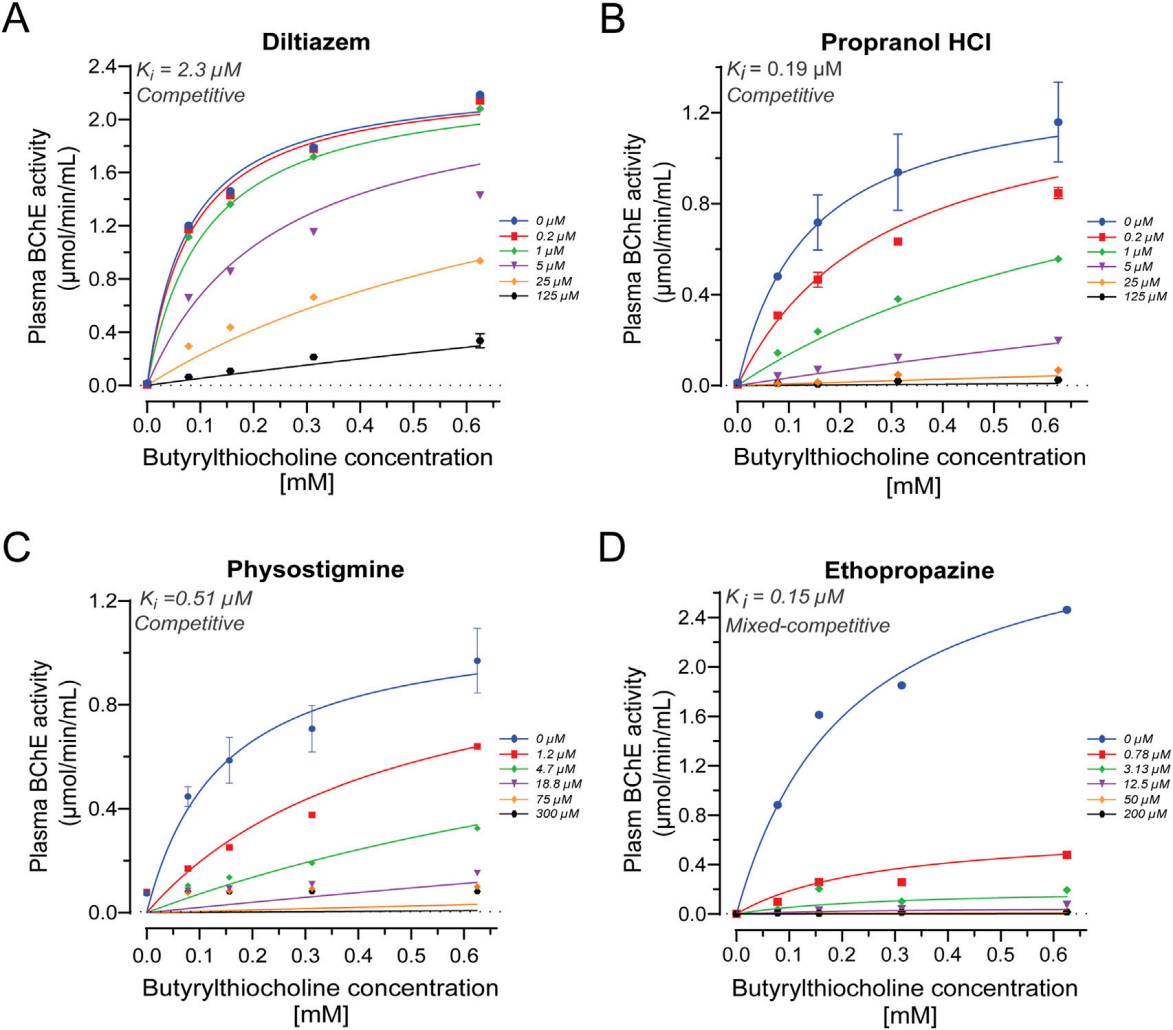
Kinetic analysis of enzyme inhibition by propranolol and diltiazem against human BChE. **(A)** Diltiazem acts as a competitive inhibitor, with nonlinear regression analysis estimating a K_i_ of 2.3 μM. **(B)** Propranolol also exhibits competitive inhibition, with a calculated K_i_ of 0.19 μM. **(C)** Physostigmine, a known non-selective cholinesterase inhibitor, shows a K_i_ of 0.51 μM for human plasma BChE based on similar nonlinear regression analysis. **(D)** Ethopropazine, a potent and selective BChE inhibitor, displays a K_i_ of 0.15 μM. These comparisons indicate that propranolol has a higher affinity for BChE than physostigmine and a comparable affinity to ethopropazine. Nonlinear regression analyses were performed using GraphPad Prism 9. Data are presented as mean ± standard deviation (SD).

To account for potential substrate concentration effects and to better understand the mechanism of inhibition, *ex vivo* enzyme kinetic analyses were performed to determine the K_i_ for each compound against BChE (Table 2). Diltiazem exhibited a K_i_ of 2.3 µM (95% CI: 1.68–3.04 µM; Figure 6A), while propranolol demonstrated a substantially lower K_i_ of 0.19 µM (95% CI: 0.15–0.23 µM; Figure 6B), indicating a higher potency. For comparison, physostigmine showed a K_i_ of 0.51 µM (95% CI: 0.28–0.91 µM; Figure 6C), and ethopropazine, a potent and selective BChE inhibitor, had a K_i_ of 0.15 µM (95% CI: 1.25–1.76 µM; Figure 6D). Moreover, the inhibition mode was also assessed, which determined that both diltiazem and propranolol exert competitive inhibition over BChE, as well as physostigmine (Figures 6A–C). In contrast, ethopropazine acts as a mixed competitor (Figure 6D).

These findings highlight the strong BChE inhibitory potential of propranolol, which is comparable to that of ethopropazine, and suggest that both propranolol and diltiazem may act as selective BChE inhibitors with limited activity against AChE.

## 4 Discussion

This study investigated the cholinesterase inhibitory properties of two FDA-approved drugs, propranolol and diltiazem, to evaluate their potential as symptomatic treatments for cognitive impairment, particularly in the context of AD and related dementias. The findings demonstrate that both compounds selectively and potently inhibit BChE, with minimal activity against AChE, suggesting a promising therapeutic profile for AD patients, in whom BChE becomes the predominant cholinesterase in the brain.

Indeed, the therapeutic relevance of BChE inhibition has gained increasing recognition in recent years. Although AChE has traditionally been the primary target in AD treatment, BChE activity is known to increase as AChE levels decline in the later stages of the disease (Mesulam M. et al., 2002; Mishra et al., 2019). Thus, the elevated expression ratio of BChE to AChE during the progression of AD suggests that it may be more advantageous to inhibit BChE, particularly in the middle and late stages of AD (Sun et al., 2024). Moreover, an increase in the uptake of radiolabelled BChE in the brain of AD mice was observed when compared to control mice, particularly in the cortical and hippocampal regions. This uptake coincides with the presence of Aβ (Rejc et al., 2021), also highlighting its importance as a target in AD treatment. The expression of BChE has also been observed in glial cells and various brain regions, including the neocortex, the amygdala, and the thalamus. This suggests a more extensive role in modulating cholinergic tone and neuroinflammation (Perry et al., 1978; Darvesh et al., 1998). Unlike AChE inhibitors, which are often associated with peripheral cholinergic side effects such as bradycardia, nausea, and hypotension (Giacobini, 2004; Geula and Nagykery, 2007), BChE inhibitors tend to have a more favourable safety profile (McGleenon and Passmore, 1999), making them attractive candidates for long-term use in elderly populations.

In this context, we found that propranolol acts as a potent and selective competitive inhibitor of BChE. Our experimental analysis yielded an IC_50_ value of 55.6 µM, which aligns well with previously reported IC_50_ values for plasma cholinesterases, ranging from 1.5 µM to 63 µM (Whittaker et al., 1982). This broad range of inhibitory potency appears to reflect propranolol’s differential affinity for various genetic variants of BChE, as compared to the wild-type enzyme. Notably, earlier studies demonstrated that atypical BChE variants exhibit reduced sensitivity to propranolol inhibition, suggesting that genetic polymorphisms significantly influence the drug’s inhibitory profile (Whittaker et al., 1981; 1982).

To further characterize propranolol’s inhibitory potency, we performed *ex vivo* enzyme inhibition kinetic analyses, which yielded an inhibition constant (Kᵢ) of 0.19 µM. This value is comparable to that of the selective BChE inhibitor ethopropazine (Kᵢ = 0.15 µM) and notably lower than physostigmine (Kᵢ = 0.52 µM), indicating strong binding affinity of propranolol to BChE.

Although our experiments utilized the racemic mixture of propranolol, previous studies have shown that the S-(−)-enantiomer exhibits 2–6-fold higher potency as a BChE inhibitor compared to the racemate, and 3–10-fold higher potency than the R-(+)-enantiomer (Whittaker et al., 1982). This enantioselectivity highlights the importance of stereochemistry in cholinesterase inhibition and suggests that the S-isomer may be responsible for the majority of the observed inhibitory activity.

However, the Kᵢ values differ substantially from the estimated IC_50_ values, which can be attributed to the substrate concentration dependency of IC_50_ measurements. In our assays, the substrate concentration was approximately 5 mM butyrylthiocholine—a level conventionally used for measuring BChE activity in biological fluids such as plasma and CSF (Darreh-Shori et al., 2006; 2011b; 2011a; 2014; Vijayaraghavan et al., 2013; Jońca et al., 2015; Kumar et al., 2016; Lana et al., 2019). At such high substrate concentrations, competitive inhibitors like propranolol and diltiazem are more readily displaced from the enzyme’s active site, resulting in higher IC_50_ values (Jońca et al., 2015). In contrast, Kᵢ is a substrate-independent constant that reflects the intrinsic binding affinity of the inhibitor for the enzyme, derived from the equilibrium between the enzyme and inhibitor. This distinction underscores the greater reliability of Kᵢ values for comparing inhibitor potency across different experimental conditions. Our findings are consistent with earlier reports describing propranolol’s inhibitory activity against plasma cholinesterases and its competitive inhibition mechanism (Whittaker et al., 1981; 1982). Importantly, the updated methodology employed in this study—incorporating nonlinear regression and model selection based on statistical criteria—may offer more predictive pharmacodynamic insights, particularly under conditions of fluctuating endogenous substrate levels. Additionally, the determination of Kᵢ provides a quantitative benchmark for comparing propranolol’s potency relative to other established cholinesterase inhibitors.

From a translational perspective, propranolol’s inhibitory activity is especially notable given its established clinical use and ability to penetrate the CNS. At physiologically relevant concentrations—approximately 0.59 µM in plasma and 10 µM in the brain tissue—propranolol was shown to reduce BChE activity by approximately 50% (Cruickshank and Neil-Dwyer, 1985). In our study, a propranolol concentration of 0.19 µM reduced BChE activity to half of its baseline level within a substrate concentration range of 0.08 mM–0.62 mM. This effect is likely to prevail *in vivo*, considering that ACh levels in the CNS typically range from 0.1 to 1 µM (Mineur and Picciotto, 2023).

Indeed, propranolol has demonstrated cognitive benefits in patients with PTSD, particularly in tasks involving visual perception, which may be partially attributable to its cholinergic modulation (Mahabir et al., 2016), and also in tasks related to memory consolidation and reactivation, ameliorating PTSD symptoms (Pigeon et al., 2022; Alharbi et al., 2024). Moreover, in individuals with dementia or AD, propranolol has been effective in alleviating behavioral and psychological symptoms, particularly in cases where other psychotropic or sedative medications have failed (Petrie, 1981; Weiler et al., 1988; Peskind et al., 2005; Summers, 2006) and its long-term use for treating essential tremor have proven its safety in chronic treatment without causing any major adverse effects (Koller and Vetere-Overfield, 1989). Nonetheless, the exact mechanism underlying the improvement is not fully understood, but it is hypothesized that a downregulation of noradrenergic β-receptor signaling might contribute to reduced emotional arousal, which is a key factor in the cognitive impairment observed in PTSD (Mahabir et al., 2016). However, there are reports that seems either to favour a cholinergic enhancing mechanism or at least a significant contribution thereof. For instance, propranolol has also been reported to alleviate symptoms in other disorders characterized by cognitive decline, such as schizophrenia, a disorder with well-established link to cholinergic system (Saint-Georges et al., 2025). The effect of propranolol treatment has been found to be comparable to those of established therapeutic agents used in standard treatment protocols (Pugh et al., 1983). The cholinergic hypothesis proposes that behavioral symptoms observed in AD, dementia, and other neuropsychiatric disorders are directly linked to a loss of cholinergic function. These symptoms—such as agitation, apathy, and psychosis—are not only present in dementia-related conditions but also in PTSD and schizophrenia. Notably, patients with psychosis exhibit elevated muscarinic M2 receptor binding in the frontal and temporal cortex, and treatment with ChEIs has been associated with symptom improvement (Lai P. et al., 2001). Previous studies have measured dopamine levels, ChAT activity, and dopamine D1 receptor density in the frontal and temporal cortices of AD patients with agitation (Cummings et al., 2024). Compared to controls, only ChAT levels were significantly reduced (Minger et al., 2000), highlighting that cholinergic dysfunction may underlie both cognitive and non-cognitive behavioral symptoms across a range of neurological and psychiatric disorders (Minger et al., 2000; Garcia-Alloza et al., 2005). In addition, these findings further support the hypothesis that the beneficial effects of propranolol in conditions such as PTSD, agitation, and schizophrenia may not be solely due to its β-adrenergic blockade, but also to its BChE inhibitory activity. This is particularly compelling considering that the S-(−)-isomer, which possesses 60 times greater β-blocking activity than the R-(+)-isomer (Howe and Shanks, 1966), is less effective in reducing symptoms of manic psychosis and schizophrenia (Gruzelier et al., 1979), suggesting that mechanisms beyond β-blockade—such as cholinesterase inhibition—may play a significant role (Whittaker et al., 1982).

Considering propranolol’s strong potency as a BChE inhibitor and its likely cholinergic-enhancing effects *in vivo*, future studies should carefully explore the possibility that propranolol’s dual pharmacodynamic actions could contribute to the observed clinical improvements, not only in this context but also in other cognitive disorders, such as Alzheimer’s and Lewy body dementia (Minger et al., 2000; Cummings et al., 2024; Saint-Georges et al., 2025).

Diltiazem has also been identified as a selective BChE inhibitor, although it exhibits lower potency compared to propranolol, with a reported Kᵢ of 2.3 µM. In humans, oral administration of 60 mg diltiazem four times daily results in a steady state plasma concentration of 222.3 ± 168.3 ng/mL (0.54 ± 0.41 µM) (Yeung et al., 1996). However, larger doses are likely to achieve higher plasma concentrations and have proven to be safe (Zelis and Kinney, 1982). This finding that diltiazem behaves as a selective BChE inhibitor is novel and to best of our knowledge has not been reported previously. Nonetheless, experimental data exist that indicates diltiazem may indirectly modulate ACh release in rats (Tsuda et al., 1994).

Diltiazem’s inhibitory effect on BChE is particularly interesting due to its additional neuroprotective mechanisms. In animal models of aluminium chloride-induced neurotoxicity, diltiazem reduced brain AChE activity, increased antioxidant enzyme levels, and improved performance in memory and learning tasks (Rani et al., 2015). These effects are likely mediated through its calcium channel-blocking properties, which help prevent intracellular Ca^2+^ overload—a key contributor to oxidative stress, Aβ accumulation, and tau hyperphosphorylation (Iqbal and Grundke-Iqbal, 2007).

Additionally, diltiazem has been shown to exert anti-inflammatory effects by modulating cytokine production, including IL-10 and IL-6 (Dubey and Hesong, 2006), further supporting its potential role in neurodegenerative disease management. Intriguingly, acetylcholine has been shown to be a key anti-inflammatory agent involved in regulation of immune cells (Rosas-Ballina and Tracey, 2009), and as such inhibition of BChE by diltiazem may increase ACh levels and/or duration of action and thereby play a key role in the observed modulation of the cytokine production.

It is also important to note that hypertension has been identified as a risk factor in most common forms of dementia, including AD. This is due to the fact that chronic hypertension can lead to neurodegeneration and cognitive decline. The utilisation of antihypertensive pharmaceuticals has been demonstrated to produce neuroprotective effects. Among these medications, calcium channel blockers have been identified as the most efficacious due to their capacity not only to reduce blood pressure but also to their previously described mechanisms regarding calcium regulation (Lyon et al., 2024). However, the potential impact of diltiazem due to its cholinesterase inhibitory effect should not be overlooked, considering that ChEIs possess a range of anti-hypertensive effects, such as lowering heart rate (bradycardia), reducing the contractile forces of the atria, and slowing the conduction velocity in both the sinoatrial and atrioventricular nodes (Nordström et al., 2013; Hu et al., 2018; Liu et al., 2019; Cavalcante et al., 2020).

Altogether, the findings of this study support the potential repurposing of propranolol and diltiazem as BChE inhibitors. Alternatively, a preferential prescription over other similar drugs to elderly at risk of dementia may prove more beneficial. Propranolol, in particular, demonstrated a strong and selective inhibitory profile, with pharmacologically relevant potency and a well-stablished safety record. It is also possible, if it is required, to substantially increase the potency of this drug through stereoisomeric purification (Whittaker et al., 1982). Diltiazem, while less potent, may offer additive or synergistic benefits through its calcium channel-blocking and anti-inflammatory effects. These dual mechanisms could be specially valuable in multifactorial conditions like AD, where cholinergic dysfunction, oxidative stress, and neuroinflammation converge to drive cognitive decline.

However, several limitations should be acknowledged. First, this study was conducted *ex vivo*, and the pharmacokinetic and pharmacodynamic properties of these compounds in the human brain may differ *in vivo*. Second, although propranolol and diltiazem are well tolerated in their approved indications, their long-term effects on cognition remain to be fully elucidated. Finally, the stereoisomeric composition of propranolol was not addressed in this study, despite evidence suggesting that its enantiomers may differ in cholinesterase binding affinity. Further studies using animal models will be essential to better elucidate the mechanisms of action and safety profiles of these compounds.

In conclusion, this study demonstrates that propranolol and diltiazem exhibit selective inhibitory activity against BChE, with propranolol showing potency comparable to one of the most potent known BChE inhibitors. These findings highlight the potential of repurposing these widely used cardiovascular drugs for the treatment of cognitive impairment in AD and related disorders. Further *in vivo* studies and clinical trials are required to explore their efficacy, safety, and mechanistic contributions to cognitive outcomes.

## Data Availability

The raw data supporting the conclusions of this article will be made available by the authors, without undue reservation.
